# Omega-3 PUFAs’ efficacy in the therapy of coronary artery disease combined with anxiety or depression: a meta-analysis

**DOI:** 10.3389/fpsyt.2024.1368007

**Published:** 2024-06-24

**Authors:** Yiwei Gui, Dongyu He, Junwei Li, Haibin Zhao

**Affiliations:** ^1^ Beijing University of Chinese Medicine, Beijing, China; ^2^ Hainan Medical University, Haikou, China; ^3^ Oriental Hospital, Beijing University of Chinese Medicine, Beijing, China

**Keywords:** omega-3 polyunsaturated fatty acids, depression, anxiety, coronary heart disease, therapy

## Abstract

**Objective:**

The evidence demonstrates that omega-3 polyunsaturated fatty acids (omega-3 PUFAs) protect the cardiovascular system and alleviate anxious or depressive situations. We conducted a meta-analysis to evaluate the effectiveness of omega-3 PUFAs in the treatment of anxiety or depressive states in patients with coronary artery disease.

**Methods:**

This meta-analysis analyzed data from randomized controlled trials to determine the efficacy of omega-3 PUFAs alone or in combination with conventional psychotropic medications in coronary artery disease patients suffering from anxiety or depression. Primary outcomes included changes in depression scores, the Beck Anxiety Inventory (BAI) scores, and the omega-3 index.

**Results:**

Included were 6 trials involving 2,570 participants. Compared to controls,omega-3 PUFAs did not improve depression or anxiety {depression: [SMD=0.09 (95% CI: -0.07, 0.26)], anxiety [BAI: SMD=0.07 (95% CI: -0.17, 0.32)]}; For the results of the subgroup analyses, analyzed by different types of depression scales, four studies used the HAMD scale [SMD=0.19 (95% CI: -0.20, 0.58)]; 5 studies used the BDI-II scale [SMD=0.01 (95% CI: -0.07, 0.09)], all of which indicated no decrease in depression scale scores; analyzed by different types of intervention, 3 studies used the omega-3 PUFAs group [SMD=0.24 (95% CI: -0.26, 0.74)]; 2 studies used sertraline + omega-3 PUFAs [SMD=-0.08 (95% CI: -0.46, 0.31)], and the omega-3 index was elevated [SMD=1.33 (95% CI: 0.18, 2.49)], suggesting that the body’s omega-3 content was indeed replenished but did not change the patient’s depressive state; analyzed by different courses of therapy, a 10-week course of therapy [SMD=0.02 (95% CI: -0.23, 0.26)] and a 12-week course of therapy [SMD=0.40 (95% CI: -0.40, 1.20)] both resulted in a lack of improvement in depressive symptoms.

**Conclusion:**

According to the available evidence, omega-3 PUFAs do not alleviate anxiety or depression in coronary artery disease patients.

**Systematic Review Registration:**

https://www.crd.york.ac.uk/PROSPERO/, identifier CRD42023391259.

## 1 Introduction

Coronary heart disease (CHD) is caused by the narrowing or occlusion of coronary arteries, resulting in ischemia, hypoxia, or necrosis of the myocardium. It has the highest death rate (1), and anxiety and depression can make it more likely that bad prognostic events will happen. A relevant summary analysis indicates that anxiety and depression increase the incidence of adverse cardiac outcomes in acute myocardial infarction prognosis by 36% (2). Treating patients with CHD combined with anxiety or depression is based on combining anxiolytic-depressants, which include selective serotonin reuptake inhibitors (SSRIs) and benzodiazepines (BZD), with cardiovascular drugs. Even so, side effects and negative effects can include granulocyte insufficiency, elevated heart rate, endocrine disturbances, and upright hypotension (3). The accompanying medical costs and poor prognosis have greatly burdened society and families, so how to effectively prevent and treat them has become a hot issue.

Current studies suggest that the combined pathological mechanisms of anxiety and depression in CHD are linked to inflammatory factors, neurotransmitters, the hypothalamic-pituitary-adrenal (HPA) axis, endocrine markers, and high-sensitivity C-reactive protein (hs-CRP) (4, 5), where the inflammatory response is a critical component of the combined anxiety and depressive disorder episodes in coronary artery disease (6). The main components of omega-3 PUFAs include alpha-linolenic acid (ALA), eicosapentaenoic acid (EPA), and docosahexaenoic acid (DHA), which can only be consumed through food and cannot be generated by the human body, with deep-sea fish oil being its most important source, and whose antidepressant effect is linked to reduced production of pro-inflammatory cytokines (7), but also participate in neuromodulatory transmission and play a positive role in depression (8); Furthermore, it possesses cardioprotective properties, such as avoiding sudden cardiac death and ameliorating heart failure (9, 10). Several trials have proven its efficacy in preventing cardiovascular disease and depression. Nevertheless, several studies do not provide significant evidence that taking omega-3 PUFA supplements improves cardiovascular disease and depression (11).

It can be seen that the efficacy of omega-3 PUFAs in treating anxiety or depression in patients with CHD is debatable, and there is no relevant meta-analysis. Therefore, the value of omega-3 PUFAs in treating anxiety or depression must be demonstrated further. We compiled the relevant published clinical research literature and hope this meta-analysis will resolve the controversy and provide clinicians and patients with new treatment options.

## 2 Materials and methods

### 2.1 Literature search

The PubMed, Cochrane Library, Embase, and Web of Science receipt databases were chosen and searched for the period from each database’s inception until February 2023. The inquiry was conducted primarily with subject phrases and free words. For instance, the terms Coronary Disease [mesh]; Coronary Diseases [Title/Abstract]; and Disease, Coronary [Title/Abstract] were used to search PubMed.

### 2.2 Inclusion and exclusion criteria

#### 2.2.1 Inclusion criteria

The included research was randomized controlled trials (RCTs); patients with coronary artery disease and anxiety or depression, whose diagnosis was based on criteria described in the literature, were required to constitute the study population, no matter their age or gender; The intervention group received omega-3 PUFAs supplements, which contained EPA, DHA, or ALA. There were no limitations on the kind, amount, duration, or frequency of the supplements, and they could be combined with conventional psychotropic medications, while the control group received a placebo; the underlying treatment measures and outcomes were measured consistently and statistically correctly in the trial and control groups; The main results obtained consisted of alterations in depression scores and the omega-3 index. The clinical trial met ethical specifications and had an exact time and place to be conducted.

#### 2.2.2 Exclusion criteria

Exclude conference abstracts, systematic reviews, case reports, animal experiments, errors and omissions in the primary outcome indicators, and the article for which accurate data were unavailable.

### 2.3 Data extraction

Two researchers individually searched and evaluated the relevant literature based on the inclusion criteria, eliminating studies that did not meet the requirements and viewing the entire text for issues that might fulfill the inclusion criteria. In cases of conflicting opinions, a consensus was formed through conversation, or a third investigator was invited to weigh in and render a decision. Only the literature with the most complete reported data was included. After the initial data extraction using the self-designed data extraction form, a secondary verification was conducted to confirm the data’s legitimacy and dependability. The specific extracts were as follows: the name of the first author, publication date, nation, sample size, gender, mean age, intervention, length of therapy, and outcome indicators. Two researchers examined the final included literature independently using the Cochrane system’s risk of bias evaluation technique for RCTs.

### 2.4 Quality evaluation

Utilizing the Cochrane Handbook for Systematic Reviewers 5.4.0 quality assessment criteria, the included RCTs’ methodology was assessed, which included randomization, allocation concealment, blinding, data bias, selection bias, reporting of results, and the presence of other tendencies. The effects of each item were evaluated as “yes” (low bias risk), “unclear,” or “no” (high bias risk).

### 2.5 Statistical analysis

Stata 15.1 was used to process the extracted information. The odds ratio (OR) was selected to characterize dichotomous variables, the standard mean difference (SMD) was used to describe continuous variables, and the 95% confidence interval (CI) was used to analyze the data estimates of the outcome indicators. The *I^2^* and P values were selected to investigate the heterogeneity between study outcomes: if *I^2^* ≤50% and P≥0.1 indicated good data homogeneity, a fixed-effects model was selected; if *I^2^* >50% and P<0.1 indicated heterogeneous data, a random-effects model was selected, and the source of heterogeneity was actively sought. Using a funnel plot and Egger’s test, publication bias was evaluated; if P > 0.05, the risk of publication bias is low; if not, publication bias may exist. A sensitivity analysis was evaluated to assess the results’ dependability.

## 3 Result

### 3.1 Study selection flowchart and result

**Figure 1:**
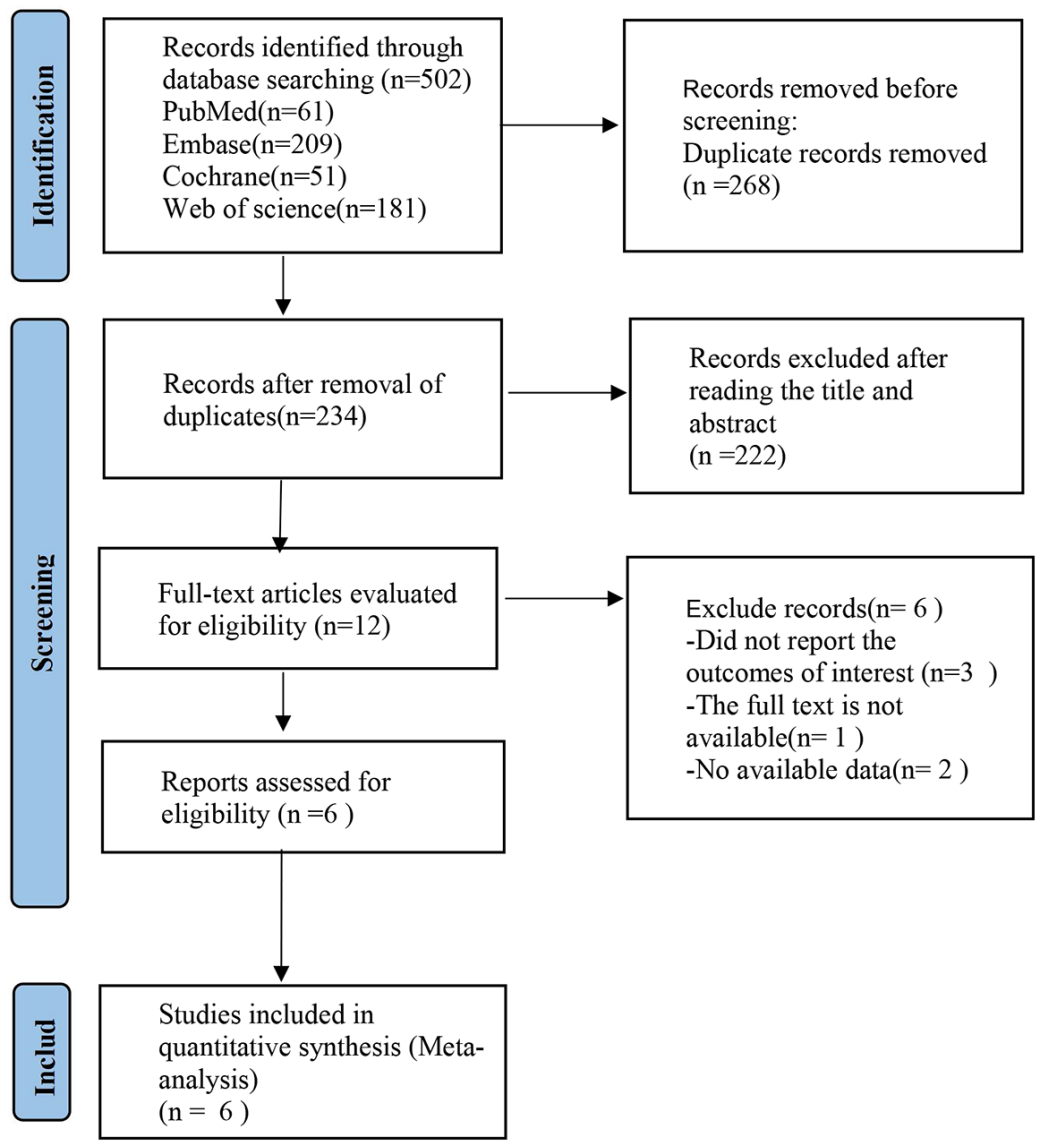
Flowchart of literature search.

The preliminary literature search yielded a total of 502 articles, of which 234 were obtained after eliminating duplicate publications. After perusing the titles and abstracts of 12 studies, 6 were included in this study after further evaluation **Figure 1**.

### 3.2 Characteristics of included studies

A total of 6 (12–17) randomized controlled trials were included. Included were 2570 patients with coronary artery disease combined with anxiety or depression; compared to the placebo group, there were 1,290 in the trial group **Table 1**.

**Table 1:** Basic characteristics of the literature.

Study	Year	Country	Sample size		Gender (M/F)	Mean age		Intervention		Treatment time	Outcome
			EG	CG		EG	CG	EG	CG		
Mazereeuw	2016	Canada	45	47	70/22	63.8	59.7	omega-3 PUFAs,1.9g/d (EPA 1200 mg/d DHA 600 mg/d)	placebo	12W	F1;F2;F3
Carney, R. M.	2010	USA	36	36	44/28	56.8	57.9	50 mg/d of sertraline +omega-3 PUFAs,2 g/day	50 mg/d of sertraline+ placebo	10w	F3;F4
Carney, R. M.	2019	USA	71	73	88/ 56	60.5	58.5	50 mg/d of sertraline +EPA,2 g/day	50 mg/d of sertraline+ placebo	10w	F3;F4;F5;
Carney, R. M.	2009	USA	62	60	81/41	58.1	58.6	50 mg/d of sertraline+omega-3 PUFAs,2g/d (EPA 930 mg/d DHA 750 mg/d)	50 mg/d of sertraline+ placebo	10w	F3;F4;F5;F6
Jane Pei-Chen Chang	2020	China	30	29	38/21	61.1	61.93	omega-3 PUFAs,3g/d (2g EPA+1g DHA)	placebo	12w	F3
Zimmer R.	2013	Germany	1046	1035	1599/482	63	64	omega-3 PUFAs,1g/d (EPA 460 mg/d, DHA 380 mg/d)	placebo	48W	F3

(F1: EPA; F2: DHA;F3: depression scores (HAMD, BDI, HDRS^12^ score, PHQ-9 score); F4: omega-3 index (% EPA+DHA in RBC); F5: BAI scores; F6: Weekly BDI- II scores).

HAMD, Hamilton rating scale for depression; HDRS^12^ score, the 12-item Hamilton Depression Rating Scale; BDI, the Beck Depression Inventory; PHQ-9 score, the 9-item Patient Health Questionnaire score.

### 3.3 Bis risk assessment map

**Figure 2:**
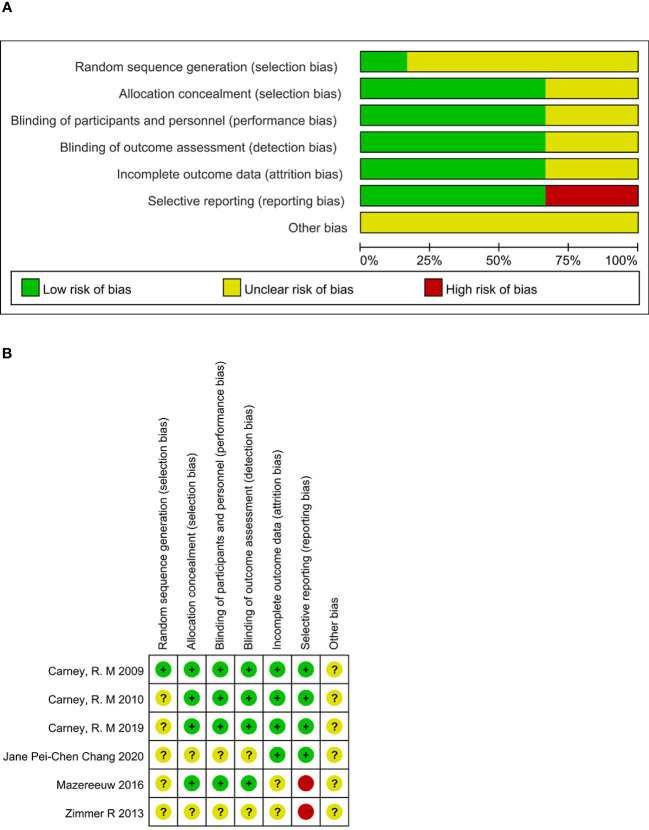
Risk of bias. **(A)** Risk of bias graph. **(B)** Risk of bias summary.

All 6 studies were RCTs; 1 article (12) mentioned that “the central office performed randomization”; 5 articles (13–17) mentioned “randomization” but did not describe the specific randomization method. Among the randomized scheme allocation hides, 4 articles (12, 13, 15, 16) mention allocation hiding. Among the blind methods, 4 articles (12, 13, 15, 16) explicitly stated that blinding was used for subjects, investigators, and outcome evaluators. 2 articles (14, 15) did not report outcome indicators in full. As shown in **Figure 2**.

### 3.4 Results of meta-analysis

#### 3.4.1 Depression scores

**Figure 3:**
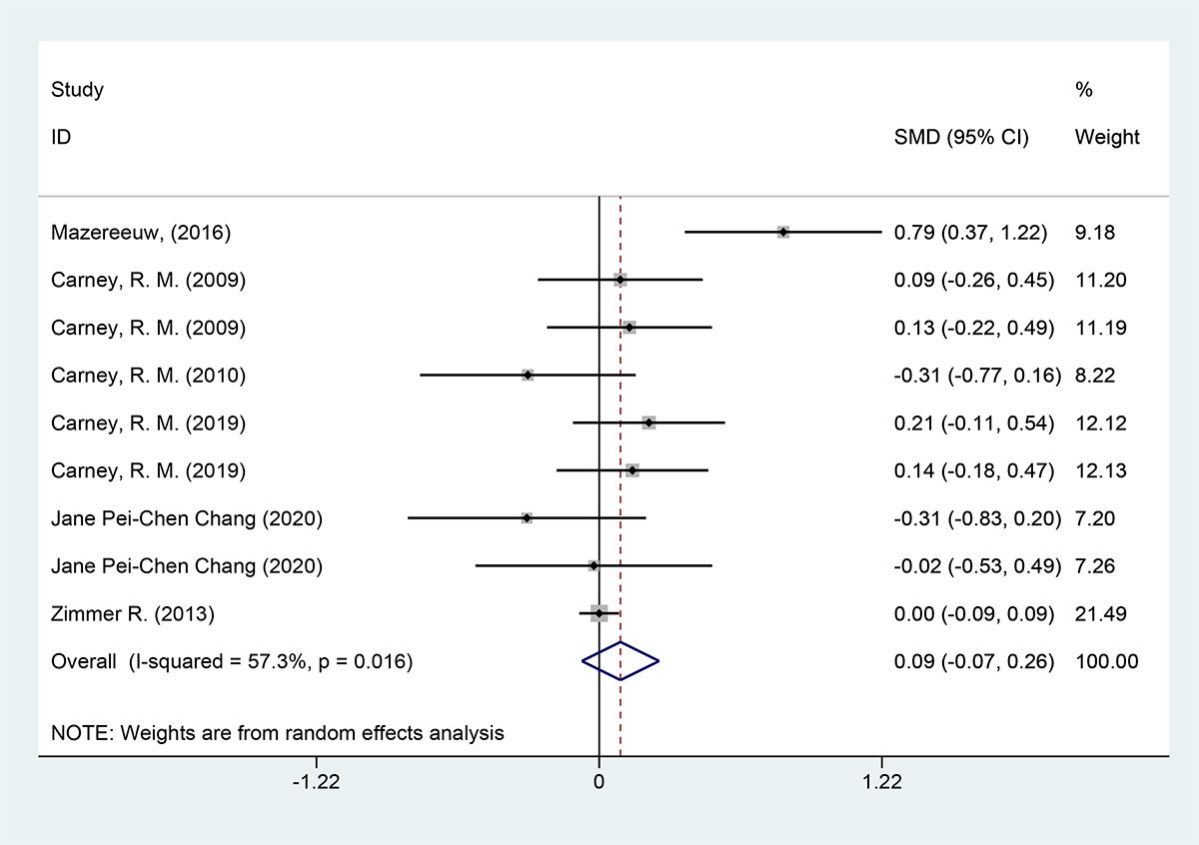
Forest plot of depression scores.

The depression scores were included in six studies, with a total of 2570 cases. They were tested for heterogeneity, which was significant across studies (P = 0.016, *I^2^* = 57.3%); employing a random effects model revealed that the test group was not able to reduce depression scale scores in contrast to the placebo group [SMD = 0.09 (95% CI: - 0.07, 0.26)] **Figure 3**.

#### 3.4.2 BAI scores

**Figure 4:**
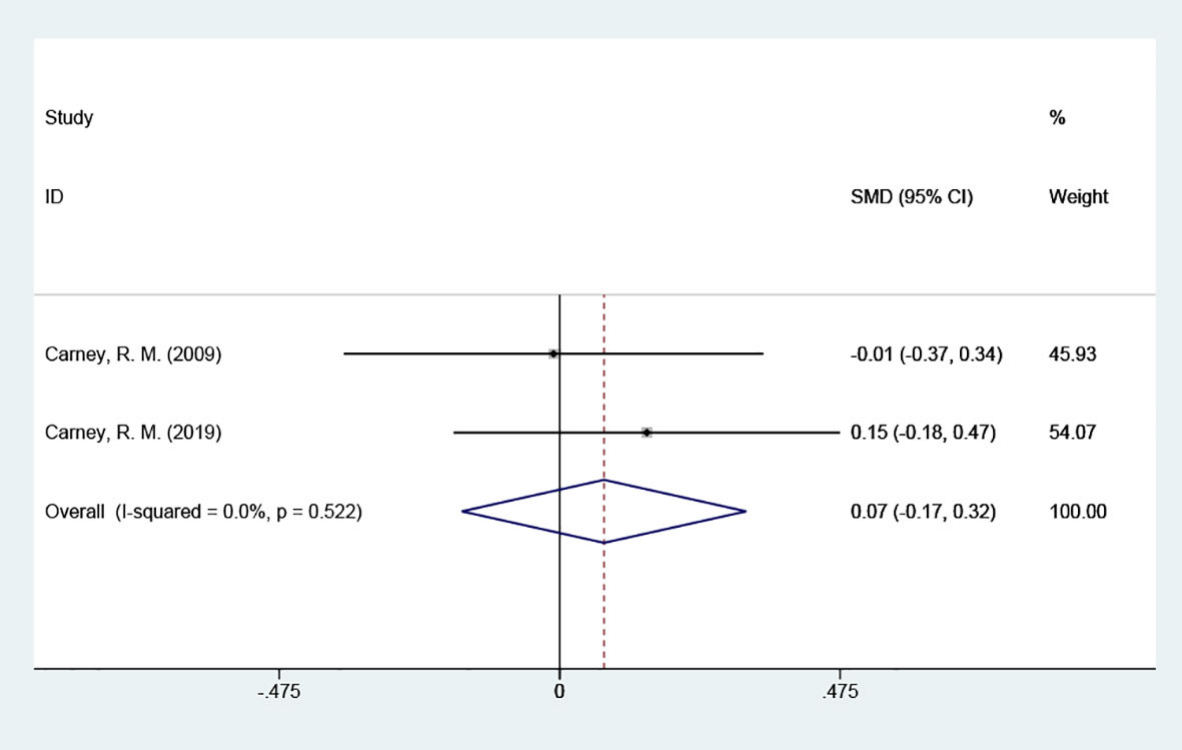
Forest plot of BAI scores.

BAI scores were included in 2 studies with a total of 266 cases, tested for heterogeneity with good homogeneity between the results of the studies (P = 0.522, *I^2^* = 0%), and using a fixed effects model indicated that it suggested that the test group could not reduce the scores of the anxiety scale in contrast to the placebo group. [SMD = 0.07 (95% CI: -0.17, 0.32)] **Figure 4**.

#### 3.4.3 Omega-3 index

**Figure 5:**
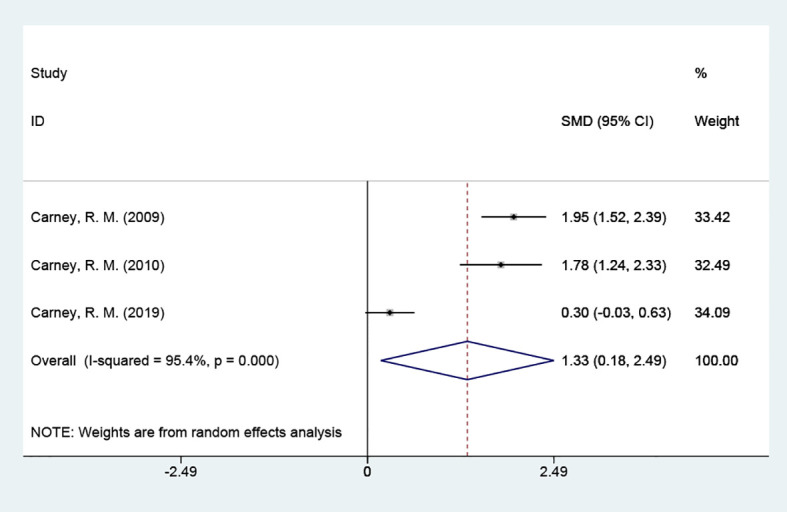
Forest plot of omega-3 index.

The omega-3 index was included in 3 studies with 388 cases and tested for heterogeneity; there was variability and heterogeneity between the groups (P = 0.000, *I^2^* = 95.4%), and using a random effects model showed that the omega-3 index was elevated. [SMD = 1.33 (95% CI: 0.18, 2.49)] **Figure 5**.

#### 3.4.4 Subgroup analysis of depression scores

##### 3.4.4.1 Grouped by different scale types

**Figure 6:**
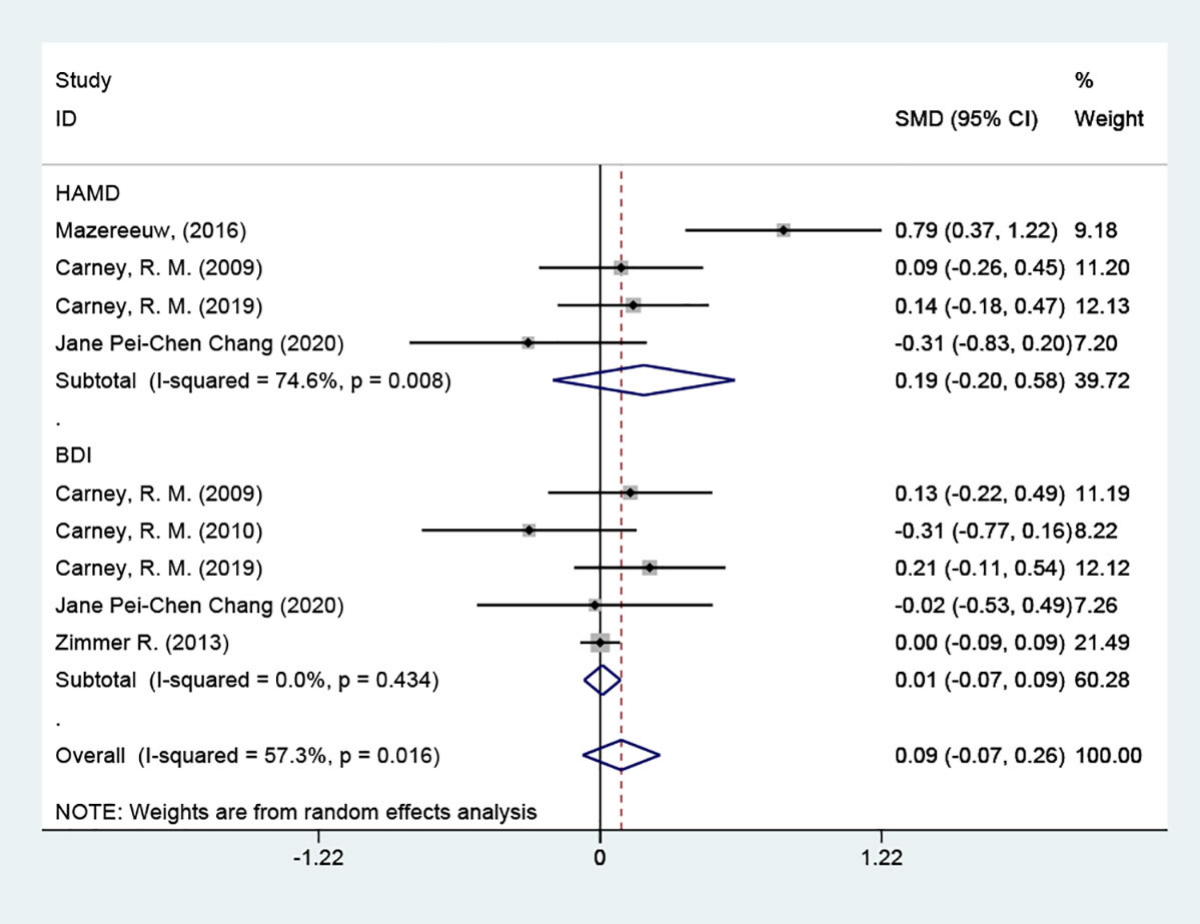
Subgroup analysis: depression scores.

4 of the studies used the HAMD scale. The results indicated that the test group was not able to reduce depression scale scores in contrast to the placebo group [SMD = 0.19 (95% CI: -0.20, 0.58)]; 5 studies used the BDI scale, and the results indicated that the test group was not able to reduce depression scale scores in contrast to the placebo group [SMD = 0.01 (95% CI: -0.07, 0.09)]. Patients with coronary artery disease did not show an improvement in their depressive symptoms. Tracing back to the original literature, different studies used different versions of depression scales (HAMD, BDI, HDRS_12_ score, and PHQ-9 score), which may be the main reason for the significant heterogeneity **Figure 6**.

##### 3.4.4.2 Grouped according to different types of intervention

**Figure 7:**
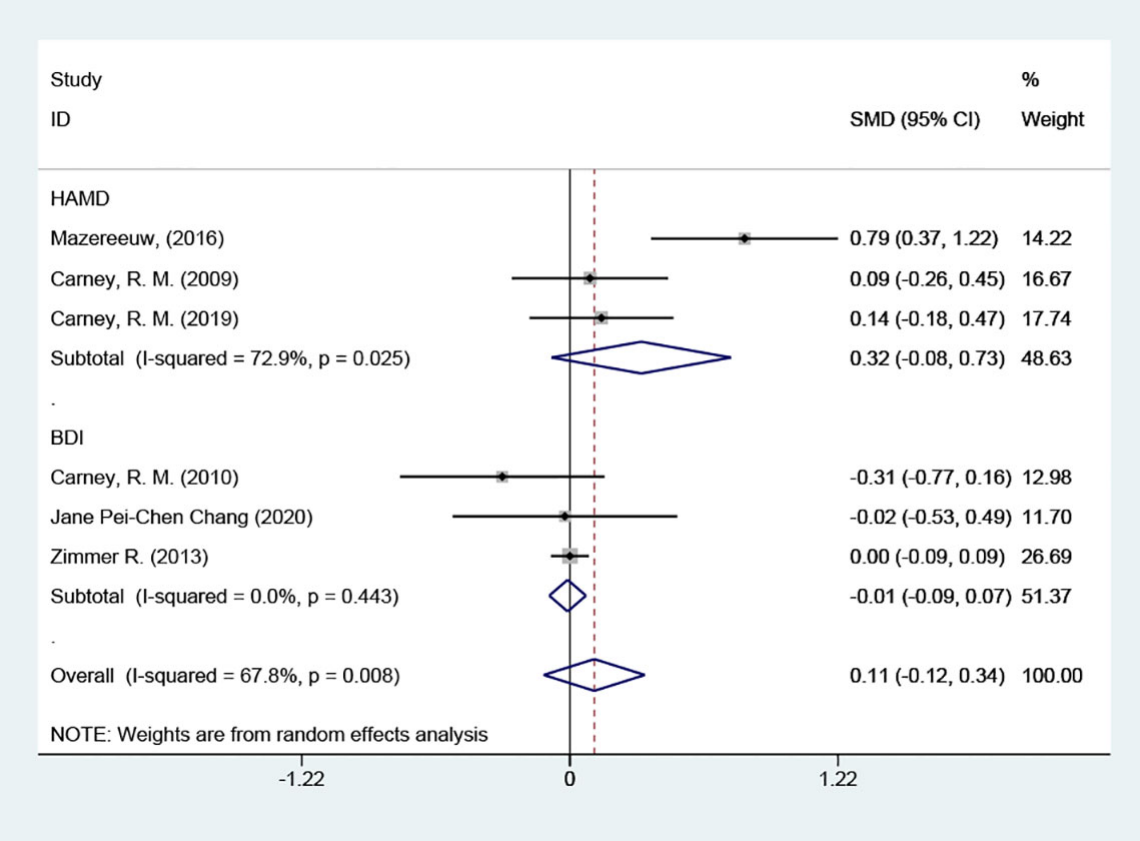
Subgroup analysis: Types of intervention.

3 of the studies used omega-3 PUFAs, and the results suggested that the omega-3 PUFAs group was not able to reduce depression scale scores in contrast to the placebo group [SMD = 0.24 (95% CI: -0.26, 0.74)]; 2 of these papers used sertraline + omega-3 PUFAs, and the results showed that, in contrast to the placebo group, it was also not able to reduce depression scale scores [SMD = -0.08 (95% CI: -0.46, 0.31)] **Figure 7**.

##### 3.4.4.3 Grouped according to the different courses of treatment

**Figure 8:**
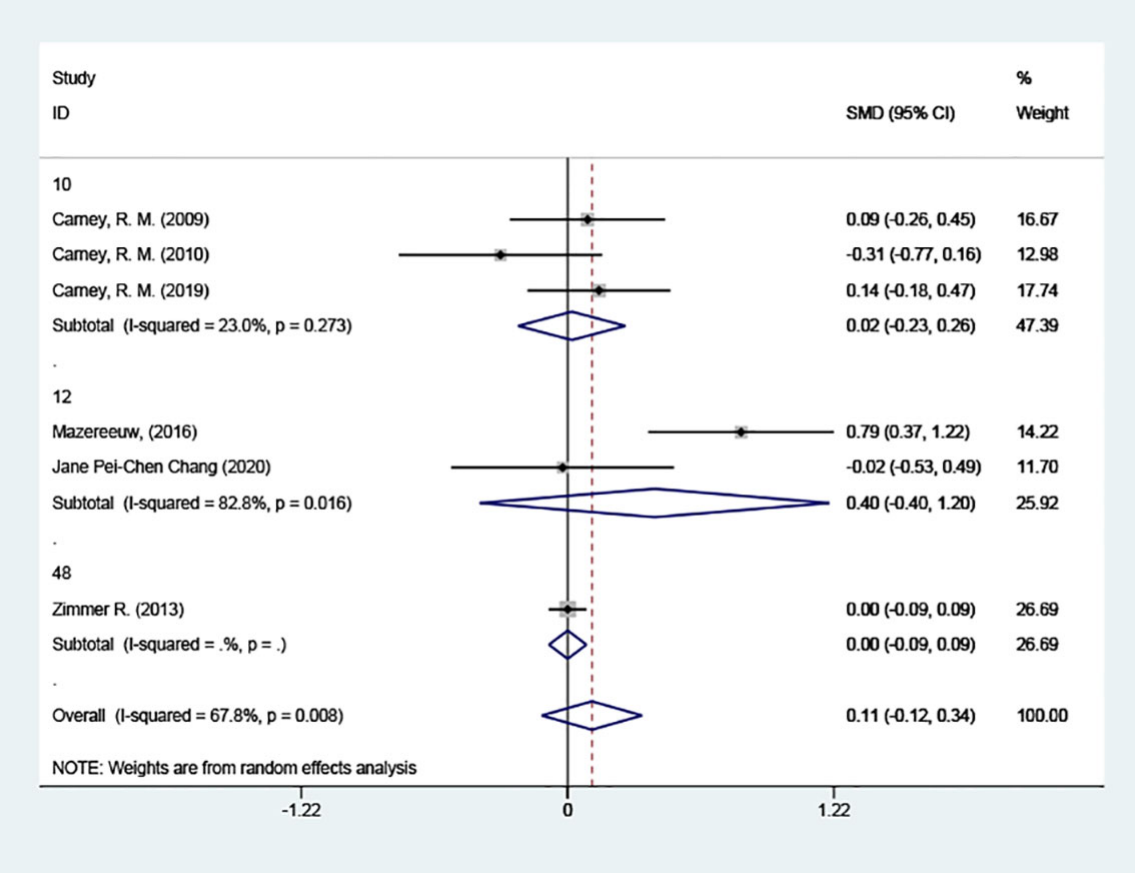
Subgroup analysis: Course of treatment.

3 of the studies had a 10-week course of treatment. In contrast to the placebo group, depression scores did not decrease after 10 weeks [SMD = 0.02 (95% CI: -0.23, 0.26)]; 2 of the articles had a 12-week course of treatment, and the results also indicated no reduction in depression scores after 12 weeks. [SMD = 0.40 (95% CI: -0.40, 1.20)] **Figure 8**.

### 3.5 Sensitivity and publication bias analysis

**Figure 9:**
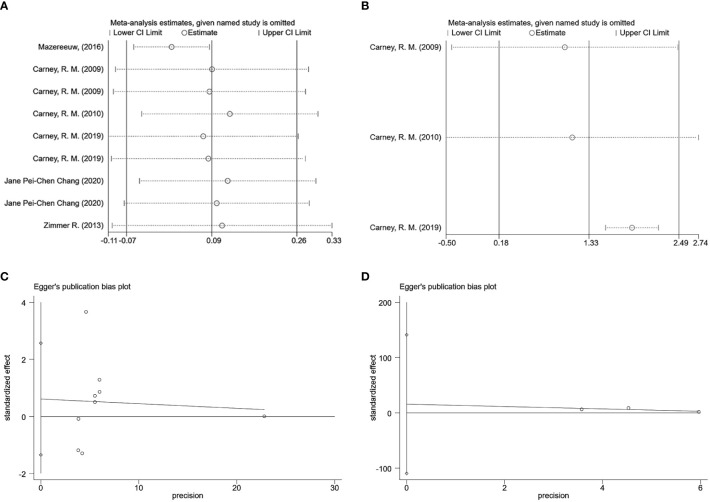
Sensitivity and publication bias analysis **(A)** Sensitivity analysis of depression scores. **(B)** Sensitivity analysis of omega-3 index. **(C)** Egger graph of depression scores. **(D)** Egger graph of omega-3 index.

Because there was still significant heterogeneity within subgroups of depression scale scores, further sensitivity analyses were conducted to reassess the combined effect values using the one-by-one exclusion method. The findings of the analysis demonstrated that effect sizes, after one-by-one exclusion from the literature, remained within the boundaries, indicating good stability of the analysis results (**Figures 9A, B**). We used Egger’s test for assessing publication bias. Using the depression scale score as an indicator, the risk of publication bias was evaluated for the included studies, p = 0.488, suggesting a higher likelihood of publication bias for this outcome indicator. Using the omega-3 index as an indicator, p = 0.355, also suggesting a higher likelihood of publication bias for this outcome indicator (**Figures 9C, D**).

## 4 Discussion

This study sought to determine whether omega-3 PUFAs could reduce depression or anxiety in people with coronary artery disease. We discovered that omega-3 PUFAs did not help coronary artery disease patients with depression and anxiety in the meta-analysis of this randomized controlled trial. The omega-3 index indicates the status of the body’s EPA and DHA levels (18), and a lower omega-3 index tends to suggest a greater likelihood of cardiovascular disease and mental illness (19). The depressive states of patients with coronary artery disease are associated with a reduced amount of EPA and DHA in the red blood cell membranes, i.e., a lower omega-3 index (20). In the results of the present experiment, the omega-3 index was elevated [SMD = 1.33 (95% CI: 0.18, 2.49)], indicating that the concentration of omega-3 PUFAs in humans was replenished, but this did not improve the anxiety or depression states of the patients. 3 of the studies used the omega-3 PUFAs group (SMD = 0.24 (95% CI: -0.26, 0.74)); 2 of the studies used the sertraline+omega-3 PUFAs group [SMD = -0.08 (95% CI: -0.46, 0.31)]; neither of which improved depressive status. Consequently, neither as monotherapy nor as an addition to medication, omega-3 PUFAs did not significantly alleviate depression in the current study.

It is highly contentious whether omega-3 PUFAs alleviate anxiety or depression among patients with coronary artery disease. During our investigation, we discovered that the effects of omega-3 PUFAs were more skewed towards the antidepressant than the anxiolytic. Thus, we will focus on their impacts on depressive states in the following. There is a typical pathologic relationship between coronary artery disease and anxiety or depression that involves inflammation, and serum levels of inflammatory factors such as CRP and tumor necrosis factor-alpha (TNF-α) are considerably higher in coronary artery disease patients with negative emotions, such as anxiety or depression, than in those without such emotions. DHA and EPA may suppress the release of inflammatory cytokines, including TNF-α, Interleukin 1 (IL-1), IL-2, and IL-6, which may have an anti-anxiety or anti-depressive effect (21). In addition, pathophysiological alterations in depression are associated with neuronal apoptosis and reduced neurogenesis. At the same time, omega-3 PUFAs may positively affect the positive effects of depression via numerous mechanisms altering nerve cell membrane stability, neurotransmitter transmission, neurogenesis, and neuroprotection (22–24). Moreover, omega-3 PUFA supplementation may improve the architecture of the brain’s white matter and decrease the severity of depressive symptoms in patients with major depressive disorder (25). Relative to patients with cardiovascular disease alone, patients with cardiovascular disease and depression had lower levels of total omega-3 PUFAs (26), a declining trend in DHA levels with increasing dysphoric moods, and lower DHA levels correlated with higher state depression severity (27). Greater consumption of omega-3 PUFAs may reduce the likelihood of depressive illness, enhance the efficacy of antidepressant medications, and alleviate mild depressive symptoms in post-infarction patients (28). However, other studies have revealed that even if supplementation with omega-3 PUFAs exhibited antidepressant advantages, they were minor and hampered by small sample sizes, and the results require further investigation (29, 30). Several studies indicate that omega-3 PUFA supplementation does not alleviate depression (31, 32).

There are several possible reasons for the controversy. First, we hypothesize that the therapeutic effect of omega-3 PUFAs on depression depends on the variety of omega-3 supplements used and the ratio of EPA to DHA. According to several studies, the favorable effects of omega-3 PUFAs on depression symptoms appear to rely more heavily on EPA than DHA (33). When EPA is used alone or as the predominant therapy modality, improvements in depression are observed (34). EPA supplementation enhances brain N-acetyl-aspartate, which works as a neuroprotective agent and can reverse brain shrinkage in severe depression (35). Enhanced treatment with EPA has a more significant antidepressant effect, with one meta-analysis placing a 50% lower limit on the proportion of EPA in omega-3 PUFAs supplemented (36). Sublette et al. recommend doses of EPA or DHA between 1000 and 2000 mg/d, with at least 60% EPA, and cannot exclude the possibility of more significant antidepressant effects with higher doses of EPA or DHA (37). Regarding the specific ratio of EPA to DHA, Song et al. determined that the optimal balance of EPA to DHA for depression was 2:1 or 3:1 (38). In the current trial, omega-3 supplements comprising EPA and DHA were administered separately or in combination at various doses and ratios, which may have impacted the results.

To be sure, omega-3 PUFAs have a positive effect on reducing cardiovascular disease risk and improving anxiety or depression in patients with coronary artery disease. The REDUCE-IT (39) trial showed that taking 4 grams of a purified form of EPA every day lowered the risk of a number of cardiovascular events, such as cardiovascular death, nonfatal myocardial infarction, and nonfatal stroke. Amin et al. (40) observed that there was a reduction in depression scale scores for each 4.5% increment in the omega-3 index among patients diagnosed with acute coronary syndrome (ACS). Haberka M et al. (41) discovered that administering low dosages of omega-3 PUFAs early on had a positive impact on lowering anxiety and depression in individuals with acute myocardial infarction. The above results show that omega-3 PUFAs can help improve anxiety or depression in people with coronary artery disease. They may also provide information and a clinical basis for the future use of omega-3 PUFAs in clinical trials and treatments for coronary artery disease combined with anxiety or depression, such as figuring out the best dosage or EPA/DHA ratio and the best way to supplement. Omega-3 PUFAs deficiency appears to play an important role in the network linking coronary heart disease, acute myocardial infarction, or other negative cardiovascular prognosis with adverse moods such as depression and anxiety, but the current research is still controversial, and longer, targeted studies are needed in the future.

And the possible synergistic benefits of mixing conventional antidepressants with omega-3 polyunsaturated fatty acids need additional research. It is unknown whether this is connected to the timing of medication administration or which antidepressant has the most significant benefit when combined with omega-3 PUFA supplements. Some studies indicate that using them as adjuvant therapy, such as in conjunction with antidepressants, is more effective than using them alone (42). However, our findings suggest that omega-3 PUFAs in combination with sertraline do not show a better therapeutic effect on depressed coronary artery disease patients.

In addition, our study found that neither a 10-week course of treatment [SMD = 0.02 (95% CI: -0.23, 0.26)] nor a 12-week course of treatment [SMD = 0.40 (95% CI: -0.40, 1.20)] was able to reduce scores on the anxiety or depression scales. This may be due to the longer time required for blood levels of omega-3 PUFAs to improve depression significantly and the lack of sufficient observation time in the trial (43). We anticipate further studies to figure out whether a longer treatment course can reduce depression scores, alleviate anxiety or depression, and reduce mortality in anxious or depressed coronary artery disease patients.

**Figure 10:**
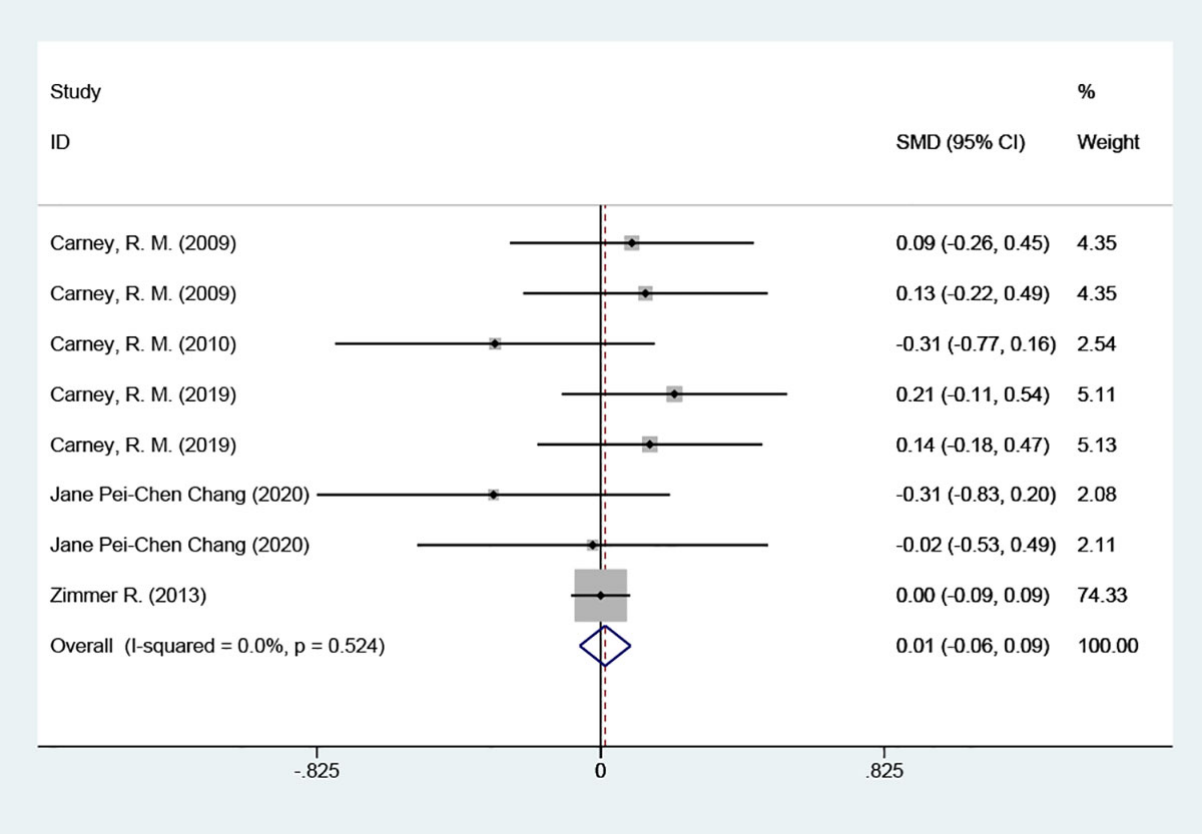
Forest plot of depression scores [After excluding Mazereeuw, G. 2016 (15)].

This study exhibited substantial heterogeneity and a high risk of bias. The magnitude of change in article heterogeneity is large after excluding this 2016 article, which is a source of significant heterogeneity **Figure 10**.

Firstly, the number of articles that met the inclusion criteria for this study was limited, and despite extensive systematic searches for topics and manual searches for relevant articles to retrieve as many eligible papers as possible, relevant studies were likely missed or excluded. Furthermore, an examination of the primary sources revealed differences in the severity of coronary artery disease and anxiety or depression among the patients in the analyzed studies, making it impossible to account for baseline levels. It’s possible that the study’s heterogeneity was caused by differences in the types and amounts of drugs given to the intervention groups, such as EPA and DHA doses and EPA/DHA ratios. There were also differences in the length of treatment and the depression scales used to measure outcomes. Although we performed detailed subgroup analyses to investigate potential sources of heterogeneity, we were unable to analyze other meaningful sources because we were limited by the original study design. Therefore, the findings should be generalized with caution. Future studies with large and representative samples are needed.

## 5 Conclusion

Current research often supplements the treatment of coronary heart disease with anxiety or depression with two main types of omega-3 PUFAs, EPA and DHA. Studies have shown that using EPA alone or in combination with DHA can significantly reduce the risk of cardiovascular disease and alleviate the symptoms of anxiety or depression. However, our research shows that taking supplements with these two main types of omega-3 PUFAs does not help with anxiety or depression in people with coronary artery disease. Neither omega-3 PUFAs nor their combination with conventional antidepressants have been shown to be effective against depression. It is important to note that ALA is one of the components of omega-3 PUFAs. However, there are limited studies on the use of ALA as an omega-3 supplement for the treatment of coronary artery disease with comorbid anxiety or depression. Therefore, there is still a need to update and include high-quality research data to support supplementation in the future, and we eagerly anticipate further clinical studies.

## Data availability statement

The original contributions presented in the study are included in the article/**Supplementary Material**. Further inquiries can be directed to the corresponding author.

## Author contributions

YG: Conceptualization, Data curation, Formal analysis, Funding acquisition, Investigation, Methodology, Project administration, Resources, Software, Supervision, Validation, Visualization, Writing – original draft, Writing – review & editing. JL: Investigation, Writing – review & editing. DH: Software, Writing – review & editing. HZ: Writing – original draft, Writing – review & editing.
